# A Perceptual Rate Control Algorithm Based on JND for Screen Content Video

**DOI:** 10.3390/s26123866

**Published:** 2026-06-17

**Authors:** Huijie Zheng, Jing Chen, Qi Lin

**Affiliations:** School of Information Science and Engineering, Huaqiao University, Xiamen 361021, China; 35534@hqu.edu.cn (H.Z.); 35520@hqu.edu.cn (Q.L.)

**Keywords:** perceptual rate control, screen content video, human visual system, just- noticeable distortion, HEVC-SCC, visual sensor network

## Abstract

The rate control algorithm is designed for natural video by default in video-coding standards. However, computer-generated screen content video (SCV) is very different from natural video captured by a camera, with many different statistical characteristics, such as sharp edges, thin lines, and flat area. This will lead to a difference in the focus of the human visual system (HVS) when viewing on-screen content video. Especially in various sensor data visualization applications such as intelligent display terminals, industrial monitoring and human–computer interaction interfaces, screen content video carries key information collected and reconstructed by image sensors, vision sensors and multimodal sensors. Its edge structures and local details directly affect the interpretation accuracy and application reliability of sensor information. Therefore, it is crucial to investigate perceptual rate control methods that integrate both video content characteristics and human visual perception properties, which possesses substantial theoretical and practical significance. In this paper, we propose a perceptual rate control algorithm for screen content video based on just-noticeable distortion (JND) which is established on the edge profile reconstruction with tolerable variations. First of all, target bit rate allocation for the frame level and CTU level is based on a perceptual weight which is calculated on the JND factor and reconstruction edge character. Secondly, under the constraint of the JND model, an intra rate-distortion (RD) model is established under the constraint of the JND model. The similarity between reference frames and reconstructed frames is taken as feedback in this model. Finally, the proposed rate control algorithm (JND–perceptual rate control (PRC)) is integrated to the existing rate control framework in High-Efficiency Video Coding–Screen Content Coding (HEVC-SCC) for improving the coding efficiency. The experimental results show that the proposed algorithm achieves better bit control precision than the platform, as well as improves the R-D performance of screen content video. In particular, compared with the HEVC-SCC reference software, the coding performance is improved by 3.09 dB on average, the bit rate is saved by 26.51% on average, and the average bit rate mismatch is within 1.159%.

## 1. Introduction

With the rapid development of multimedia communication and computer technology, screen content video generated by computers has emerged. Especially with the large-scale popularization of intelligent sensing technology, SCV has become a core data carrier for collecting, transmitting and visualizing data from image sensors, visual sensor networks and industrial monitoring sensors. In recent years, SCV applications have gradually penetrated daily life and various sensor-based scenarios, such as video conferencing relying on visual sensors for image acquisition; online education involving screen sharing of teaching content and transmission via sensing devices; game livestreaming with real-time screen capture and push through sensor networks; and industrial monitoring where on-site data is converted into SCV by image sensors for real-time transmission. Unlike conventional camera-captured video, screen content video (SCV) [1] has numerous distinctive properties. In the spatial domain, screen content video has a large number of flat areas, repeated patterns, sharp edges and limited color types. In the temporal domain, adjacent frames can be completely different from each other due to the lack of physical limitations of the camera. In other words, screen content video contains mutant frames and still frames. This characteristic leads to bit rate fluctuations during SCV transmission in sensor networks, increasing the energy consumption burden on low-power sensor nodes. However, traditional video-coding standards are designed for the characteristics of natural video by default. In response to these features, the encoding standard for screen content video has been expanded from HEVC [2,3] and is called HEVC-SCC [4], which adds four new advanced encoding tools to improve the efficiency of encoding screen content video, including Intra Block Copy (IBC) [5], Palette Mode (PLT) [6], Adaptive Color Transform (ACT) [7] and Adaptive Motive Vector Resolution (AMVR) [8]. However, the above are all improvements in encoding, and further improvements are likely needed in rate control. Rate control (RC) is an essential part of the encoder, which is beneficial for efficiently utilizing bandwidth resources and improving transmission efficiency. Rate control mainly establishes a mathematical model for the bit rate R and quantization parameter (QP) relationship. It ensures the encoded bit rate matches the target rate. In general, RC consists of two core steps. The first is bit allocation, which adopts a hierarchical coding structure to allocate appropriate bit rate for a group of pictures (GOP), frame, and coding tree unit (CTU) respectively. The second is bit control, which adjusts the encoding parameters to make the actual bit rate close to the pre-allocated bit rate by establishing the rate-distortion (RD) model. As the HEVC coding structure becomes flexible and coding tools become diversified, the accuracy of the RD model is required to be higher. The R-λ model [9] has replaced the R-Q model [10] as the default RD model in coding standards. However, the R-λ model still has many shortcomings, such as neglecting the characteristics of video content and the human visual system. In view of this, many outstanding scholars put forward many improved algorithms. In [11], the mean absolute difference (MAD) is replaced by the sum of absolute transform differences (SATDs) to guide the bit allocation at the CTU level and improve the accuracy of in-frame bit rate control. Driven by the trade-off between the order of the rate-distortion model and the difficulty in estimating the parameters of the rate control (RC) model, Liao et al. [12] proposed a high-order rate-distortion model for video coding as well as the corresponding quantization parameter decision scheme. Zhao et al. [13] utilize an adaptive Canny edge detector and a dual-threshold scheme to accurately extract the spatial–temporal content complexity of video sequences, and propose a frame-level target bit allocation method. Chen et al. [14] proposed an efficient method for end-to-end scale-adaptive video coding, which can convert sparsely distributed rate-distortion points into densely distributed ones, thus improving the coding performance. Guo et al. [15] proposed the optimal bit allocation at the frame level and GOP level by using the information of the encoded frames in the previous GOP and the time R-D dependence between different GOPs. In ref. [16], a CTU-level code rate control optimization decision is proposed to minimize the sum of CTU-level expected distortion under a given code rate constraint, a two-stage bisection-based method, and R-D performance is improved.

The above rate control methods do not take into account the subjective perceptual performance of video coding. Therefore, Zhao et al. [17] proposed a perceptual rate control method based on the structural similarity index (SSIM), which was used to measure the distortion for bit allocation and construct the rate-distortion model. However, in recent years, some researchers have pointed out that the SSIM does not reflect human visual characteristics well, since the sensitivity of human vision to different regions of video depends on physiological and psychological factors. Meddeb et al. [18] designed a bit rate control method based on Region of Interest (ROI), which classifies each coding unit of ROI described in the video frame and then allocates target bit and improved the visual quality of reconstructed video. Wei et al. [19] proposed a bit rate control method based on visual saliency, which measured the CTU-level distortion through the visual saliency algorithm, and at the same time carried out bit allocation at the image level to reduce bit fluctuation and improve the perceptual quality of video coding. Feng et al. [20] proposed a HEVC code rate control algorithm based on visual perception. This method considers the subjective quality and motion characteristics of the CTU level to construct the bit allocation module, which improves the rate-distortion performance of video coding to a certain extent. Zeng et al. [21] proposed a bit rate control method based on perceptual sensitivity, and constructed a bit allocation module considering the complexity of spatial texture perception and time-domain motion activity to improve the perceptual quality of reconstructed video. A content-aware rate control [22] scheme for HEVC based on static and dynamic saliency detection is proposed. By allocating more bits to improve the quality of the salient region and fewer bits to the non-salient region, the CTU-level rate control perception distortion is improved. Zhou et al. [23] proposed a HEVC sensing rate control method based on the minimum tolerable distortion threshold, which allocated bits through pixel-level weight factors and constructed a rate control model for coding parameter estimation. However, these methods are all aimed at traditional natural content videos.

Due to screen content and natural video having different characteristics, the improvement strategy of the bit rate control algorithm for screen content video is mainly aimed at its characteristics. Chen et al. [24] employ a pre-analysis procedure to obtain content information characterizing the complexity of SCV, and guide frame-level target bit allocation. Accordingly, they propose effective schemes for target bit allocation and model parameter updating. Sanchez et al. [25] sequentially allocate bits to the frame level and CTU level according to motion and texture characteristics, then approximate the R-D and R-QP curves of each block through a set control point and Random Sample Consensus (RANSAC), refit the relationship between R-D and R-QP for screen content video, and perform parameter estimation. Esmaeeli et al. [26] established a new video-content-based intra R-D model by considering the intra prediction modes unique to Screen Content Coding (SCC), such as Intra Block Copy (IBC), and proposed a frame-level bit allocation method based on Proportional–Integral–Derivative (PID). Lin et al. [27] extracted the spatial and temporal features of screen content videos, and then constructed a spatial–temporal feature model to guide adaptive bit allocation. Chen et al. [28] proposed a screen content rate control model based on 3D gradient guidance. By leveraging the unique spatiotemporal characteristics of screen videos, they established a spatiotemporal feature extraction scheme using a 3D gradient filter, and simultaneously extracted spatiotemporal features for guiding bit allocation. Yang et al. [29,30] proposed a content-based video rate control algorithm for screen content. After classifying CTUs by considering the content characteristics of coding frames, the rate-distortion model was constructed respectively, which improved the rate-distortion performance of video coding. However, this method did not fully consider the inter-frame correlation. Wang et al. [31] proposed a frame-level rate control method for SCV, which further divided the frames in SCV into key frames (KFs) and non-key frames (NKFs) based on inter-frame correlation and assumed reference decoder. However, current SCC rate control methods do not consider the perceptual characteristics of videos.

### Related Work Summary

Based on the above literature investigation, four key research gaps are summarized as follows:

(1) Existing HVS-inspired perceptual rate control algorithms (Refs. [17,18,19,20,21,22,23]) are developed for natural videos and fail to conduct targeted optimization for the prominent strong-edge characteristics inherent to SCV.

(2) Though the existing SCV-specific rate control approaches (Refs. [24,25,26,27,28,29,30,31]) possess content-adaptive capability, none of them incorporate explicit human visual system models such as JND.

(3) The limited existing JND-based rate control works (Ref. [23]) are tailored for pixel-domain natural videos and cannot accommodate the characteristics of SCV, such as coexistence of large flat uniform regions and sharp textual edges.

(4) To the best of our knowledge, no prior research has integrated pixel-domain JND modeling, edge profile reconstruction, edge-feature-driven perceptual weighting, and SSIM-based R-λ feedback into a unified rate control framework dedicated to SCV.

So, in this paper, we combine the actual needs of sensor application scenarios, and take into account the fact that the human visual system is interested in the edge features of screen content video, such as text and graphics, and that the luminance, contrast and structural information in screen content video can affect the visual quality of video coding, so we propose a perceptual bit rate control algorithm for screen content video based on just-noticeable distortion (JND) for the human visual system. The main contributions are as follows: (1) For the first time, a pixel-domain JND model, designed for screen content video characteristics, is used to construct the bit rate control model. (2) The perceptual characteristics of the screen content video are added as a reference basis for the allocation of target bits at the frame level and CTU level, and the bit rate is adaptively allocated according to the video content. (3) Measuring the structural similarity between the reference frame and the reconstructed frame under the JND model to guide the perceptual rate-distortion model (i.e., R-λ model) of Lagrange factor to estimate and update parameters. The remainder of this paper is organized as follows. Section 2 introduces the JND model-building process of screen content video in detail. Section 3 describes the bit allocation method and rate-distortion model optimization process based on the JND model. Section 4 presents the experimental results that verify the effectiveness of this research method. Finally, the conclusion of this paper is detailed in Section 5.

## 2. JND Model Construction and Perceptual Feature Extraction

Just-noticeable distortion refers to pixel-level changes below the minimum threshold of visibility that cannot be perceived by the HVS. Meanwhile, the edge is an important feature that human eyes can perceive. In particular, screen content video contains a large number of edge features, such as text, chart borders, etc., which are thinner than the edges of natural video. Therefore, we adopt a pixel-domain JND model [32] for SCV and reconstruct the edge of screen content images. The details will be discussed in this section.

### 2.1. Edge Parameter Model

**Figure 1:**
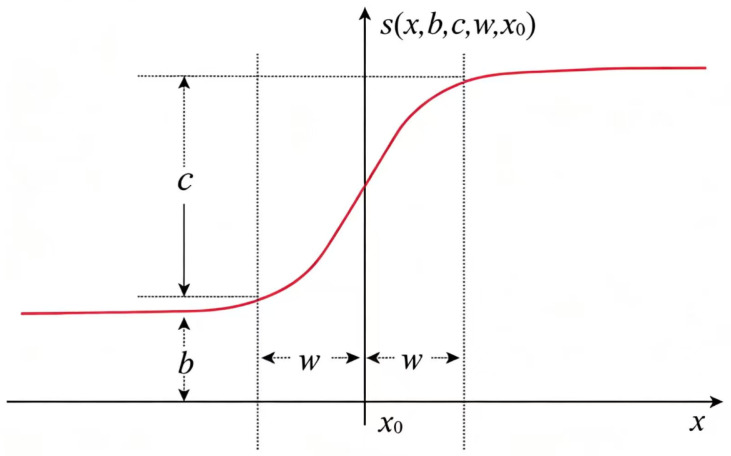
One-dimensional smooth step edge structure model.

Both in human vision and computer vision, edges describe important image structures and form an important factor in restoring the original scene structure. The purpose is to model and analyze the edge to extract relevant image information. In general, the edge is a point in a one-dimensional signal, or a curve in a two-dimensional intensity surface or image. The unit step function can be used for describing the one-order edge model.

(1) $$u\left(x;b,c,x_{0}\right)=c\cdot U\left(x-x_{0}\right)+b,$$

where U(·) denotes the unit step function and $x_{0}$ indicates the edge center. In fact, the step edges are not present in actual images, although they are in screen content videos with lots of sharp edges. Moreover, the real edge of SCV can be viewed as a smooth unit step edge, following [33,34]. The Gaussian function is used to smooth the step edge model.

(2) $$s\left(x;b,c,w,x_{0}\right)=u\left(x;b,c,x_{0}\right)⊗g\left(x;w\right)=b+\frac{c}{2}\left[1+\mathrm{erf}\left(\frac{x-x_{0}}{w\sqrt{2}}\right)\right],$$

(3) $$g\left(x;w\right)=\frac{1}{w\sqrt{2\pi }}\exp\left(-\frac{x^{2}}{2w^{2}}\right),$$

where erf(·) is the error function and *w* is the standard deviation of the Gaussian function, which can be regarded as the edge width reflecting the edge structure. By the above description, any edge profile can be decomposed into luminance, contrast and structure controlled by parameters *b*, *c* and *w*, respectively. As shown in Figure 1, the parameter *b* determines the base strength of the edge and *c* reflects the contrast strength of the edge. A higher *c* corresponds to stronger edges. The edge structure is controlled by *w*. The smaller *w* is, the sharper the edge profile.

The next task after fitting the edge model is to estimate these parameters. Firstly, the actual response of the edge model is obtained by convolving the Gaussian partial derivative function $g^{\prime }\left(x;w_{d}\right)$ with the edge parameter model. The subscript *d* indicates the derivative form of the Gaussian function, and $w_{d}$ is the standard deviation of the derivative Gaussian kernel, controlling the smoothing scale for edge detection.

(4) $$\begin{array}{cc} e_{1D}\left(x;c,w,w_{d}\right) & =s\left(x-x_{0};b,c,w,x_{0}\right)⊗g^{\prime }\left(x;w_{d}\right)\\ \; & =\frac{c}{\sqrt{2\pi (w^{2}+w_{d}^{2})}}\exp\left(-\frac{{\left(x-x_{0}\right)}^{2}}{2(w^{2}+w_{d}^{2})}\right) \end{array}\;,$$

(5) $$g^{\prime }\left(x;w_{d}\right)=-\frac{x}{w_{d}^{3}\sqrt{2\pi }}\exp\left(-\frac{x^{2}}{2w_{d}^{2}}\right).$$

Then, multi-point parameter estimation mentioned in the literature [33] is adopted here. Using the following three measures of this response, $e_{1}=e\left(0;c;w;w_{d}\right)$, $e_{2}=e\left(a;c;w;w_{d}\right)$ and $e_{3}=e\left(-a;c;w;w_{d}\right)$, we have

(6) $$w=\sqrt{\frac{a^{2}}{\ln\left(\frac{e_{1}^{2}}{e_{2}e_{3}}\right)}-\sigma_{d}^{2}},$$

(7) $$c=e_{1}\cdot \sqrt{\frac{2\pi a^{2}}{\ln\left(\frac{e_{1}^{2}}{e_{2}e_{3}}\right)}}\cdot {\left(e_{2}/e_{3}\right)}^{\frac{1}{4a}},$$

(8) $$b=s\left(x_{0}\right)-\frac{c}{2},$$

where *a* denotes the sampling distance, normally set to 1.

In order to describe the edges of the image, the one-dimensional edge model is extended to the two-dimensional edge model. The mathematical expression of two-dimensional smooth step edge model is as follows.

(9) $$s_{2D}\left(x,y;b,c,w,\theta \right)=b+\mathrm{erf}\left(\frac{x\cdot \cos\theta +y\cdot \sin\theta }{w\sqrt{2}}\right).$$

**Figure 2:**
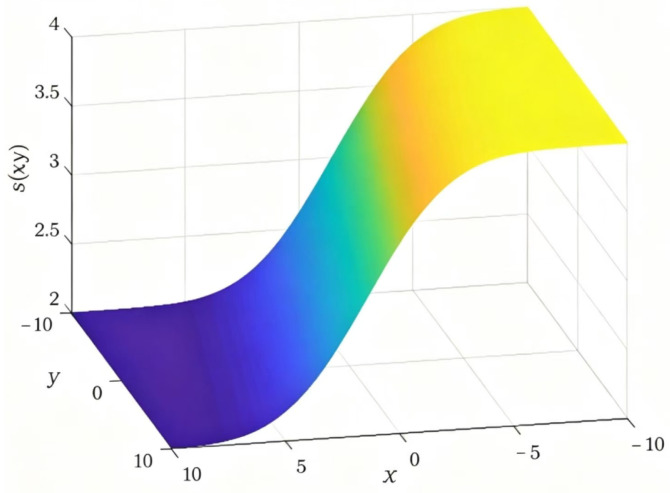
Two-dimensional smooth step edge structure model.

It can be seen from the above formula that the edge of the image can be depicted in three-dimensional space, as shown in Figure 2. The x–y plane represents the image’s pixel spatial positions, and the vertical axis stands for pixel luminance.

To detect the two-dimensional edge, the partial derivative of the two-dimensional Gaussian function in the *x* and *y* directions is used. It can be shown that

(10) $$\begin{array}{cc} e_{x}\left(x,y;c,w,\sigma \right) & =s_{2D}\left(x,y;b,c,w,\theta \right)⊗\frac{\partial }{\partial x}g_{2D}\left(x,y;\sigma \right)\\ \; & =\cos\theta \cdot e(x\cos\theta +y\sin\theta ;c,w,\sigma ) \end{array}\;,$$

(11) $$\begin{array}{cc} e_{y}\left(x,y;c,w,\sigma \right) & =s_{2D}\left(x,y;b,c,w,\theta \right)⊗\frac{\partial }{\partial y}g_{2D}\left(x,y;\sigma \right)\\ \; & =\sin\theta \cdot e(x\cos\theta +y\sin\theta ;c,w,\sigma ) \end{array}\;,$$

(12) $$g_{2D}\left(x,y;\sigma \right)=\frac{1}{2\pi \sigma^{2}}\exp\left(-\frac{x^{2}+y^{2}}{2\sigma^{2}}\right),$$

Two-dimensional edge detection operator is obtained as follows:

(13) $$\begin{array}{cc} e_{2D}\left(x,y;c,w,\sigma ,\theta \right) & =e_{x}\left(x,y;c,w,\sigma ,\theta \right)\cdot {\vec{l}}_{x}+e_{y}\left(x,y;c,w,\sigma ,\theta \right)\cdot {\vec{l}}_{y}\\ \; & =\sqrt{e_{x}^{2}\left(x,y;c,w,\sigma ,\theta \right)+e_{y}^{2}\left(x,y;c,w,\sigma ,\theta \right)} \end{array}\;,$$

To simplify the calculation, it can be expressed as

(14) $$e_{2D}\left(x,y\right)=|e_{x}\left(x,y\right)|+|e_{y}\left(x,y\right)|,$$

Furthermore, the direction of the smoothed gradient is equal to the direction of the edge:

(15) $$\tan\theta =\frac{|e_{y}\left(x,y\right)|}{|e_{x}\left(x,y\right)|},$$

The edge parameters are further calculated as follows:

(16) $$w=\sqrt{\frac{1+\tan^{2}\theta }{\ln\left(\frac{e_{1}^{2}}{e_{2}e_{3}}\right)}-\sigma_{d}^{2}},$$

(17) $$c=e_{1}\cdot \sqrt{\frac{2\pi (1+\tan^{2}\theta )}{\ln\left(\frac{e_{1}^{2}}{e_{2}e_{3}}\right)}}\cdot {\left(\frac{e_{2}}{e_{3}}\right)}^{\frac{1}{4\sqrt{1+\tan^{2}\theta }}}.$$

**Figure 3:**
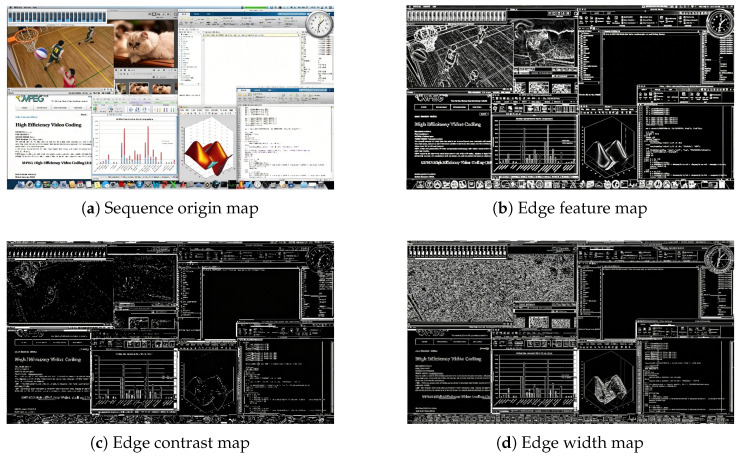
Feature extraction for “BasketballScreen” sequence.

Through the above edge model, the edge features, edge contrast and edge width mapping of the screen content video sequence are obtained, as shown in Figure 3. It can be seen from the figure that the adopted edge model can extract edge features of screen content and video well.

### 2.2. Masking Effect of Screen Content Images

According to the first subsection, we know that the edge parametric model has three main parameters *b*, *c* and *w*, corresponding to luminance adaptation, contrast masking, and edge structure, respectively. Therefore, edge reconstruction can be achieved by modifying these three parameters, and also by deriving their visibility thresholds to obtain the JND of the edge pixel set.

#### 2.2.1. Luminance Adaptation

Traditional luminance adaptive models typically rely on the N×N background luminance to derive visibility sensitivity. This approach is used to calculate the background luminance because natural content videos usually consist of areas with uniform texture. However, this property does not always apply to screen content videos. Compared to natural content video, image areas in SCVs contain larger uniformly flat areas and sharp edges. Therefore, edge pixels need to be separated from the pixels in the image region when determining the background brightness.

**Figure 4:**
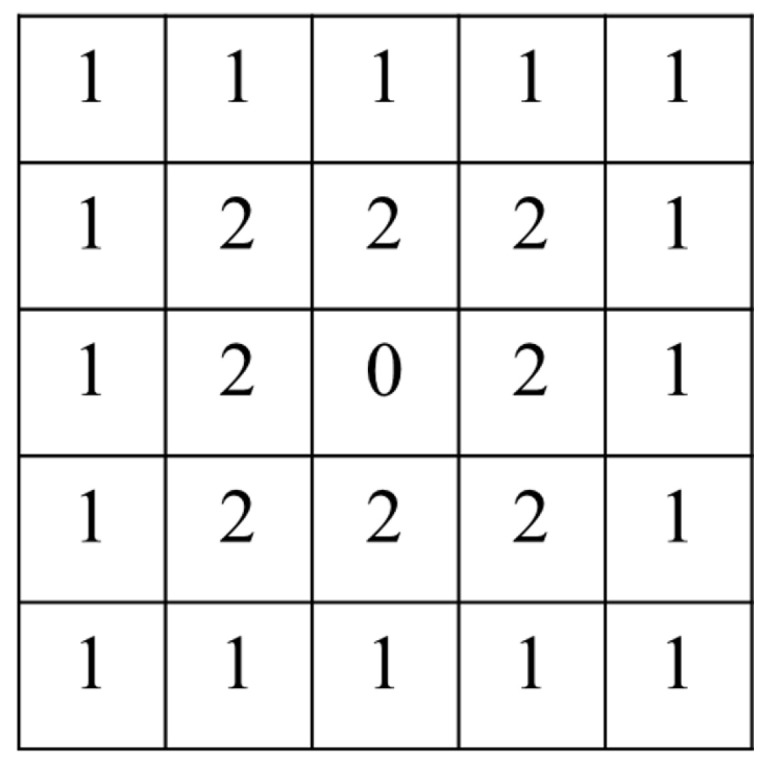
Average background brightness filtering kernel for non-edge pixel sets.

With the edge detection in edge modeling described above, pixels in screen content videos can be divided into two categories—edge pixel sets and non-edge pixel sets—and different ways of measuring luminance masking are used. According to the one-dimensional notation, the location of the pixel is assumed to be *p*, and for the non-edge pixel set the background luminance is calculated within the 5×5 window [35] shown in Figure 4. It is important to note that edge pixels are excluded from the calculation of background luminance. And when *p* belongs to the edge pixel set, the average luminance along the edge contour is I(p)=b+c/2, and the luminance masking effect formula is calculated as follows.

(18) $$T_{lum}\left(p\right)=\left\{\begin{array}{cc} \alpha_{1}\cdot \left(1-\sqrt{\frac{I(p)}{127}}\right)+\beta , & \mathrm{if}\;I(p)\leq 127\\ \alpha_{2}\cdot \left(I\left(p\right)-127\right)+\beta , & \mathrm{otherwise} \end{array}\right.\;,$$

where the parameters $\alpha_{1}=17$, $\alpha_{2}=3/128$, and β=3. The threshold 127 is the midpoint of the 8-bit grayscale range, partitioning luminance into low and high regions to match the human visual system’s nonlinear masking characteristics.

#### 2.2.2. Edge Contrast

The human visual system is more sensitive to image contrast, which is more pronounced in screen content videos, because the relative change in luminance conveys important information to the human visual system [36]. In the literature [37], a visible threshold for visible edge contrast changes was derived, and this framework has been shown to be an effective model with the following contrast masking effect function.

(19) $$T_{con+}\left(p\right)=\frac{1+f_{th}}{1-f_{th}}\cdot c\left(p\right),$$

(20) $$T_{con-}\left(p\right)=\frac{1-f_{th}}{1+f_{th}}\cdot c\left(p\right),$$

where $T_{con+}\left(p\right)$ and $T_{con-}\left(p\right)$ denote the contrast masking thresholds for positive and negative contrast increments respectively. c(p) is the edge contrast. And $f_{th}=0.14$ is an empirical parameter from HVS experiments [37], quantifying the asymmetric sensitivity of human vision to contrast changes.

#### 2.2.3. Edge Structure Distortion Sensitivity

Edge width *w* reflects the structure of the edge contour and the sharpness/blur of the image. The probability of blur distortion detection is determined by the edge width and the threshold at which the human visual system can mask the blur [38]. However, setting a constant threshold on the edge width may not be suitable for screen content videos because two high-contrast edge profiles within a screen content video may have significantly different widths. Instead, here we investigate the relatively varying visibility thresholds Δw

(21) $$\Delta w=w_{t}-w,$$

where $w_{t}$ is calculated by parameter estimation based on the distorted screen content video. In this experiment, Δw is set at around 0.1.

### 2.3. JND Model for SCV

**Figure 5:**
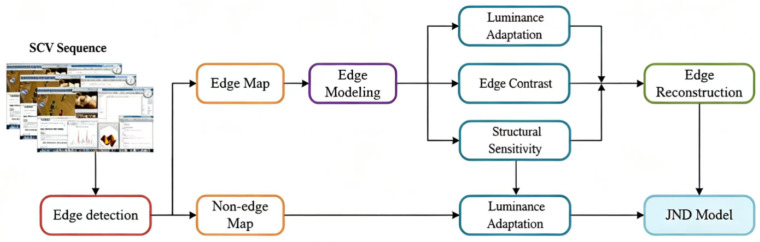
The JND model estimation process.

As shown in Figure 5, the overall JND model for screen content videos encompasses pixel classification, multi-dimensional masking effect calculation, and final threshold fusion. The JND model proposed in this paper is a multi-factor, edge-aware model. It is built on four factors that together capture the major masking effects of the HVS on screen content: (1) Luminance adaptation ($T_{lum}$): The HVS is less sensitive near the two ends of the luminance range and most sensitive in the mid-range. (2) Edge contrast masking ($T_{con}$): The visibility threshold of an edge depends on its contrast, with the asymmetric sensitivity quantified by $f_{th}$. (3) Edge structure distortion sensitivity ($T_{str}$): A sharp edge tolerates only a small width change Δw. (4) Pixel-domain classification: Pixels are partitioned into edge and non-edge sets, with the edge set using the full multi-factor JND and the non-edge set using luminance adaptation alone. This combination is what makes the JND model appropriate for screen content with both flat areas and sharp edges.

Specifically, the SCV sequence is processed by edge detection to partition pixels into an edge pixel set and a non-edge pixel set. For edge pixels, three masking effect visibility thresholds (luminance adaptation, edge contrast and structural sensitivity) are derived based on the edge parameter model established in Section 1, while non-edge pixels only consider the luminance adaptation masking threshold. Then, these thresholds are integrated to construct the unified JND model for SCVs. The details of each component are elaborated in the following sections.

Firstly, dividing the SCI into edge pixel sets and non-edge pixel sets, the luminance adaptive visibility thresholds for edge pixels *p* have

(22) $$T_{elum}\left(p\right)=s_{2D}\left(p;T_{lum}\left(p\right)+b,c,w\right)-s\left(p;b,c,w\right),$$

From the above equation, it can be seen that the luminance adaptive visibility threshold is determined by $T_{lum}\left(p\right)$. Similarly, we can obtain the pixel-edge contrast masking effect threshold.

(23) $$\begin{array}{c} T_{econ}\left(p\right)=\min\{|s_{2D}\left(p;b,T_{con+},w\right)-s_{2D}\left(p;b,c,w\right)|,\\ \;|s_{2D}\left(p;b,T_{con-},w\right)-s_{2D}\left(p;b,c,w\right)\left|\right\} \end{array}\;,$$

Here the minimum value is to ensure that the appropriate perceptibility threshold is obtained. The nonlinear Additivity Masking Mode (NAMM) [39] of $T_{elum}\left(p\right)$ and $T_{econ}\left(p\right)$ is fused to obtain the non-structural distortion sensitivity threshold at the edge of the pixel, calculated as follows:

(24) $$T_{nstr}\left(p\right)=T_{elum}\left(p\right)+T_{econ}\left(p\right)-C_{nstr}\cdot \min\left\{T_{elum}\left(p\right),T_{econ}\left(p\right)\right\},$$

where $C_{nstr}$ is a constant, in the range of $0<C_{nstr}<1$. It describes the degree of overlap between $T_{elum}\left(p\right)$ and $T_{econ}\left(p\right)$. Relatively, the structure distortion sensitivity threshold for the pixel edge is calculated as follows:

(25) $$T_{str}\left(p\right)=\left|s_{2D}\left(p;b,c,w+\Delta w\right)-s_{2D}\left(p;b,c,w\right)\right|,$$

The overall edge profile JND model for SVCs is received by fusing the non-structured distortion sensitivity and the structural distortion sensitivity threshold for edge pixels, which is also the edge pixel JND factor. It is calculated as follows.

(26) $$T_{e}\left(p\right)=T_{nstr}\left(p\right)+T_{str}\left(p\right)-C_{e}\cdot \min\left\{T_{str}\left(p\right),T_{nstr}\left(p\right)\right\},$$

In our experiment, both $C_{nstr}$ and $C_{e}$ are 0.2.

For screen content videos, edge detection is performed based on edge modeling to partition pixels into an edge pixel set $S_{E}$ and non-edge pixel regions. For non-edge pixel levels, only the luminance masking visibility thresholds are considered; by integrating the JND thresholds of the edge pixel set, the final JND model for the screen content image is constructed as follows:

(27) $$\mathrm{JND}=\left\{\begin{array}{cc} T_{e}\left(p\right), & p\in S_{E}\\ T_{lum}\left(p\right), & \mathrm{otherwise} \end{array}\right.\;.$$

## 3. Proposed Rate Control Algorithm

**Figure 6:**
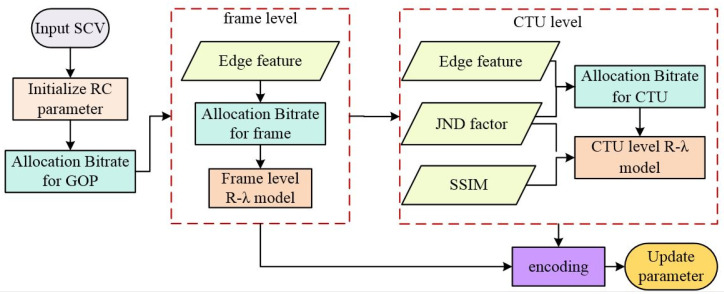
The proposed JND-PRC algorithm flow block diagram.

The RC algorithm mainly consists of two steps: target bit allocation, and parameter estimation and update. This section presents a detailed discussion on the allocation of target bits for both frames and coding tree units (CTUs) based on the perceptual complexity weight derived from the pixel-level edge just-noticeable distortion (JND) model. Furthermore, a perceptual rate control mathematical model is constructed under JND constraints, with its parameters estimated and updated by measuring the deviation in edge feature similarity of screen content videos. The framework of the proposed algorithm is depicted in Figure 6, which intuitively elaborates how the JND weight acts on each stage throughout the entire rate control pipeline. The flowchart is split into five sequential processing steps: (1) calculate the full-frame JND map and edge feature coefficients at the pre-analysis stage; (2) realize GOP and frame-level target bit allocation by deploying JND-weighted perceptual importance for hierarchical bit assignment; (3) perform CTU-level target bit allocation by introducing the block-wise JND weight $W_{\mathrm{JND}}\left(i\right)$ to distribute bits for individual coding tree units; (4) iteratively estimate the parameters of the R-λ model guided by SSIM feedback; (5) derive the final quantization parameter QP from the perceptual Lagrange multiplier $\lambda_{\mathrm{JND}}$.

In the previous section, the JND model of SCVs and JND factors by fusing the edge model parameter thresholds has been obtained. Next, the improvement of the rate control algorithm is discussed in detail.

### 3.1. Perceptual Bit Allocation

At the frame level, the perceptual weight of the current encoded frame in the GOP is calculated by considering the edge feature factor, and the code rate is assigned to the current frame.

(28) $$Tar_{curframe}=\left(Tar_{GOP}-Act_{codeframe}\right)\times \left(1-\varepsilon \right)+0.5,$$

where $Tar_{GOP}$ indicates the GOP-level target bits, obtained from the target bits of the video sequence in the encoding profile. $Act_{codeframe}$ denotes the actual bits consumed by the encoded frames in the GOP. ε is the frame-level bit allocation adjustment factor, obtained from the perceptual weight of the current frame. In a low-latency coding structure; this can be calculated by

(29) $$\varepsilon =\left\{\begin{array}{cc} PW_{cur}+0.01, & \mathrm{if}\;(0.8<PW_{cur}\leq 1)\\ 1.05\times PW_{cur}+0.01, & \mathrm{if}\;(0.35<PW_{cur}\leq 0.8)\\ 1.3\times PW_{cur}+0.01, & \mathrm{if}\;(0<PW_{cur}\leq 0.35) \end{array}\right.\;,$$

where $PW_{cur}$ denotes the perceived weight of the current encoded frame in the encoded GOP, which is calculated as follows.

(30) $$PW_{cur}=\frac{EF_{curframe}}{EF_{un-codeframe}},$$

where $EF_{curframe}$ denotes the edge feature factor of the current encoded frame, which is predicted from the reference frame of the current encoded frame, and is calculated as follows.

(31) $$EF_{curframe}=|e_{x}\left|+\right|e_{y}|,$$

where $e_{x}$ and $e_{y}$ denote the edge feature factors along the X and Y directions of the current frame, respectively.

At the CTU level, in addition to considering the edge feature factor, the difference in JND factor between blocks is also an important factor affecting the bit rate allocation. For the currently encoded CTU, the edge feature perception factor is the sum of the absolute weighted values of the pixel-level edge factors obtained along the X and Y directions, respectively.

(32) $$EF_{curCTU}=e_{CTUx}+e_{CTUy},$$

(33) $$e_{CTUx}=\sum_{i=1}^{N}\left|e_{xpi}\left(x,y\right)\right|,$$

(34) $$e_{CTUy}=\sum_{i=1}^{N}\left|e_{ypi}\left(x,y\right)\right|,$$

The bit allocation method is determined by the perceived weight of the current CTU, and the perceived weight is calculated as follows:

(35) $$PW_{curCTU}=\frac{EF_{curCTU}}{EF_{uncodeCTUs}},$$

where $EF_{curCTU}$ is the edge feature perception factor of the current CTU and $EF_{uncodeCTUs}$ represents the edge feature perception factor of the unencoded CTU in the encoded frames. Compared with natural content videos, there are a large number of sharp edges and flat areas in screen content videos. The impact of the perceptual sensitivity of the human visual system on HEVC-SCC is considered, and thus the bit allocation strategy is modified. Therefore, using the edge feature perception factor, the complexity weight of the current block is defined as [40]

(36) $$CW_{curCTU}=\frac{EF_{curCTU}/\left(W_{CTU}\times H_{CTU}\right)}{EF_{curframe}/\left(W\times H\right)},$$

where $W_{CTU}$ and *W* are the widths of the current CTU and frame, respectively. $H_{CTU}$ and *H* are the heights of the current CTU and frame, respectively. The CTUs are classified into complex, continuous and simple CTU blocks based on the complexity weight of the encoded CTUs. The CTUs are grouped into complex CTU blocks, which contain more perceptual information and require more bits to be allocated, and simple CTUs, which require fewer bits, with the weighting factors for the different CTUs defined as

(37) $$F\left(CW_{curCTU}\right)=\left\{\begin{array}{cc} 3, & \mathrm{if}\;(CW_{curCTU}>1.2)\\ 1.25, & \mathrm{if}\;(0.5<CW_{curCTU}\leq 1.2)\\ 0.5, & \mathrm{otherwise} \end{array}\right.\;,$$

Finally, the target bits of the current CTU are calculated

(38) $$\begin{array}{cc} Tar_{CTU}\left(i\right)= & \left(\frac{B_{curleft}\times PW_{curCTU}\times F\left(CW_{curCTU}\right)}{W_{JND}\left(i\right)}+0.5\right)\\ \; & +\frac{\sum_{codedCTUs}\left(Tar_{codedCTU}-Act_{codedCTU}\right)}{SW} \end{array}\;,$$

where $Tar_{CTU}\left(i\right)$ presents the *i* th CTU target bit currently encoded, and $B_{curleft}$ represents the remaining bits of the current encoded frame. $Tar_{codedCTU}$ and $Act_{codedCTU}$ represent the encoded CTU target bit and the actual bit, representing the size of the sliding window set to 40 in this chapter. $W_{JND}\left(i\right)$ represents the weight of the JND, the distortion threshold that can be tolerated by the human eye encoding the current CTU. The larger the value is, the greater the distortion degree that can be tolerated by the current CTU is, and the smaller the target bit allocated is. Its calculation is as follows:

(39) $$W_{JND}\left(i\right)=\frac{JND_{curCTU}/\left(W_{CTU}\times H_{CTU}\right)}{JND_{curframe}/\left(W\times H\right)}.$$

### 3.2. Parameter Estimation and Update

In the HEVC-SCC reference platform, after bit allocation at the GOP level, frame level and CTU level, coding parameters are determined by the model and expressed as

(40) $$\lambda_{scc}=\alpha \times bpp_{JND}^{\beta },$$

where α and β are model parameters related to the input video characteristics. bpp denotes the coding bits per pixel, which is calculated as follows:

(41) $$bpp_{JND}=\frac{Tar_{CTU}\left(i\right)}{W_{CTU}\times H_{CTU}},$$

where $Tar_{CTU}\left(i\right)$ is the target bit of the current CTU, and $W_{CTU}$ and $H_{CTU}$ are the widths and heights of the current CTU respectively. In order to improve the performance of SCV sensing coding, this method proposes a parameter estimation method based on the just-noticeable distortion perceptual rate control model of human eye perceptible distortion. Perceptual bit rate control model parameters $\lambda_{JND}$ and quantization parameters $QP_{JND}$ are calculated as follows:

(42) $$\lambda_{JND}=k\times \left(T_{JND}\times SSIM_{JND}\times \lambda_{scc}\right),$$

where *k* is the model parameter related to SCV content characteristics, when the encoding frame is P frame, k=0.0155 and for B frame, k=0.19. The average visibility threshold factor $T_{JND}$ of the current encoded frame is calculated as follows:

(43) $$T_{JND}=\left\{\begin{array}{cc} \frac{JND_{curframe}}{W\times H}+2.2, & \mathrm{if}\;JND_{curframe}/\left(W\times H\right)<4.7\\ \frac{JND_{curframe}}{W\times H}, & \mathrm{if}\;4.7<JND_{curframe}/\left(W\times H\right)<6.67\\ 6.4, & \mathrm{if}\;6.67<JND_{curframe}/\left(W\times H\right)<7\\ 6.6, & \mathrm{otherwise} \end{array}\right.\;.$$

The quantization parameter $QP_{JND}$ is obtained through the Lagrange parameter and the calculation formula is given in [41] as follows:

(44) $$QP_{JND}=4.2005\times \lambda_{JND}+13.7122+0.5.$$

$SSIM_{JND}$ is the current encoding frame based on the distortion similarity perceptible by human eyes, which is shown as follows [42].

(45) $$SSIM_{JND}=\frac{2EF_{r}EF_{d}+c_{1}}{EF_{r}^{2}+EF_{d}^{2}+c_{1}}.$$

where *r* represents the reference frame, *d* represents the reconstructed frame, and $c_{1}$ is to avoid division by zero.

## 4. Analysis of Experimental Results

To verify the effectiveness of our method, the JND-PRC algorithm proposed was integrated into the HEVC-SCC test model HM-16.10+SCM-8.0 and Low Delay was used as the coding structure for comparison with the HM baseline, without rate control, by the method in [30]. In order to facilitate the description, the name of all rate control algorithms is simplified. Firstly, if hierarchical bit allocation in the test model HM-16.10+SCM-8.0 is set as 2, the rate control method is named “SCM-8.0-A”, which is the adaptive ratio bit allocation. What is more, if hierarchical bit allocation in the test model HM-16.10+SCM-8.0 is set as 1, the rate control method is named “SCM-8.0-H”; in this method, the bit allocation scheme at frame level is conducted with the ratio introduced in [27]. Meanwhile, hierarchical bit allocation in the test model HM-16.10+SCM-8.0 is set as 0, the method is named “SCM-8.0-NH”, and the target bits for each frame are allocated equally. The approach proposed in our paper is denoted as “JND-PRC”. The test sequence uses the three categories recommended in the JCT-VC conference [31], such as text and graphics with motion (TGM), mixed content (M), animation (A), which are in the format YUV444. To ensure the comparability and reproducibility of the experiment, we have uniformly selected 160 frames as the test sequence. The coding parameters for all the screen content test sequences are shown in Table 1. In order to show that the JND-PRC proposed algorithm performs well over a wide range of code rates, the initial QPs 22, 27, 32 and 37 are set to encode without code rate control, and the final code rates obtained from the four QPs are used as the four target code rates for code rate control. The rest of the encoding parameters in the experiments were used as the default parameters in the configuration file. In this paper, the performance of the proposed perceptual rate control algorithm for HEVC-SCC is comprehensively evaluated from four perspectives: bit rate mismatch, perceptual rate-distortion performance and subjective visual quality.

### 4.1. Bit Rate Accuracy Comparison

Bit rate mismatch (BRM) is a perspective to evaluate the rate control algorithms for video coding, which is defined as

(46) $$\mathrm{BRM}=\frac{|R_{tar}-R_{act}|}{R_{tar}}\times 100\%,$$

where $R_{tar}$ and $R_{act}$ represent the target and actual bit rates, respectively. The smaller the BRM is, the more accurate the target bit rate can be achieved, which enables the method to be more adaptable to corresponding network condition and applications. The BRM comparison of the proposed algorithm with the original rate control in HEVC-SCC is performed on SCM-8.0 when hierarchical bit allocation is set to 0, 1 and 2, respectively, under LDB coding structures. Specifically, all test sequences are encoded with fixed QPs by the original SCM-8.0 when hierarchical bit allocation is set to 0, 1 and 2 respectively without rate control. It can be seen from Table 2 that our method’s average BRM is smaller than SCM-8.0 in different hierarchical bit allocation conditions for the most of the test videos. The average bit rate error of our algorithm is 1.528%, 2.370% and 1.159% when hierarchical bit allocation is set to 0, 1 and 2, respectively. In other words, our algorithm achieves better rate control precision as the whole.

### 4.2. R-D Performance

In this subsection, the effectiveness of the proposed method is evaluated by the rate-distortion performance [43]. Bjøntegaard-Delta Bit Rate (BD-BR) and Bjøntegaard-Delta PSNR (BD-PSNR) indicators are adopted, where BD-BR indicates the percentage bit rate savings of the two methods at the same objective quality level. BD-PSNR measures the improvement of video quality at the same bit rate. As shown in Table 3, BD-PSNR and BD-BR are calculated by comparing with the algorithms SCM-8.0-NH, SCM-8.0-H and SCM-8.0-A for SCVs with different characteristics including TGM, M and A. When SCM-8.0-NH is used as an anchor, the mean BD-PSNR of JND-PRC-NH is 4.07 dB. In comparison with SCM-8.0-H, the average BD-PSNR of JND-PRC-H is 3.05 dB. What is more, compared with SCM-8.0-A, the BD-PSNR of JND-PRC-A is 3.09 dB on average. Among different SCV types, TGM has the highest average quality improvement under the same bit rate, reaching 4.42 dB. Meanwhile, the JND-PRC saves bit rate a great deal, which for the BD-BR is −34.85%, −24.46% and −26.51% when hierarchical bit allocation is set to 0, 1 and 2, respectively.

**Figure 7:**
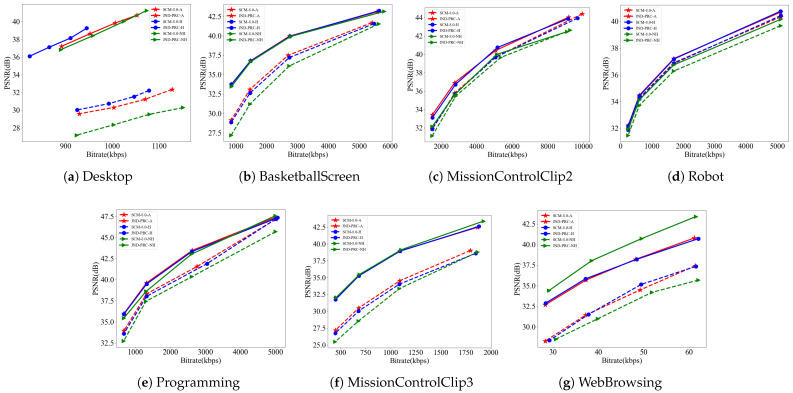
R-D curve comparison.

Furthermore, the rate-distortion (R-D) curves are plotted in Figure 7, where the solid lines correspond to our proposed JND-PRC algorithm and the dashed lines represent the baseline SCM-8.0 scheme. The plotted curves reveal that JND-PRC achieves superior coding performance against SCM-8.0. These results verify the rationality and effectiveness of the JND-based bit allocation strategy.

In addition, to verify the performance advantages of the JND-PRC algorithm, this paper conducts a rate-distortion performance comparison with Yang et al. [30] and Lin et al. [27]. From the data in Table 4, it can be seen that when SCM-8.0-H is used as the reference anchor point, JND-PRC outperforms the average BD-PSNR in the literature [30] by 2.18 dB, outperforms the average BD-PSNR in the literature [27] by 1.15 dB, and reduces the average BD-BR by 24.21%. Compared with the method in the literature [27], the proposed JND-PRC achieves competitive or better performance on most test sequences, delivering a higher average BD-BR reduction. However, on specific sequences such as Console, MissionControl2, and Robot, the BD-YPSNR improvement of JND-PRC is lower than that of [27]. This observation demonstrates the complementary strengths of edge–perceptual and temporal–statistical approaches to screen content rate control, indicating that neither method holds universal superiority over the other. A detailed discussion and in-depth analysis of this phenomenon is provided in Section 4.4.

### 4.3. Visual Quality Performance

**Figure 8:**
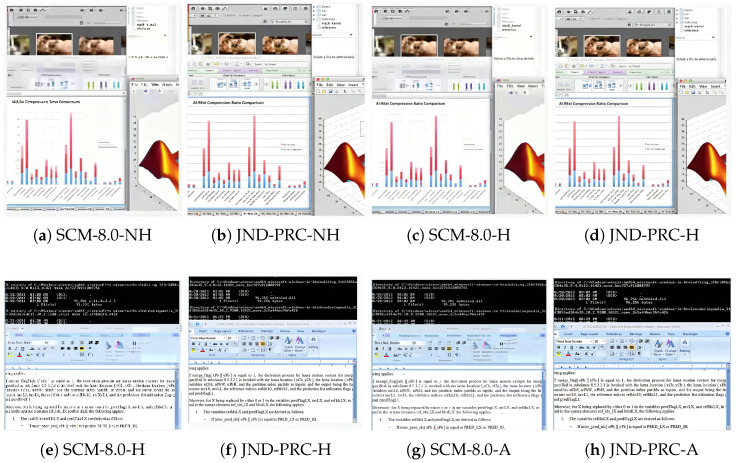
Visual quality comparison for SCM8.0 and the proposed JND-PRC.

It is well known that in addition to using objective indicators to evaluate video quality, the visual quality perceived by human eyes has practical significance. Figure 8 shows the visual quality comparison with the anchor at QP = 32 for the first reconstructed picture of “BasketballScreen” and “Desktop”. The results demonstrate that the subjective quality of the proposed JND-PRC algorithm is superior to SCM 8.0 in both image area and text area, when hierarchical bit allocation is set to 0, 1 and 2, respectively.

### 4.4. Discussion and Limitation Analysis

This section analyzes the compatibility of JND-PRC with VVC/VTM and its performance limitations on certain test sequences. The core design of JND-PRC is compatible with VVC. Its JND model, built upon pixel-level edge features and human visual characteristics, requires no modification for migration. The frame- and CTU-level bit allocation follows the GOP–Frame–CTU hierarchical structure adopted by VVC, so the perceptual weights $PW_{cur}$ and $CW_{curCTU}$ can be directly integrated. In addition, VVC retains the HEVC R-λ model for rate-distortion optimization, and our λ and QP optimization based on $SSIM_{JND}$ feedback works well under this framework. In summary, the core algorithmic logic of the JND-PRC method can be adapted to VVC.

Compared with the method in the literature [27], the proposed JND-PRC algorithm achieves lower BD-YPSNR gains on specific sequences. On the Console sequence with slow-moving content and slight frame changes, the edge feature factor varies little, weakening perceptual weight discrimination. For MissionControl2, insignificant differences in JND thresholds between flat and textured regions impair CTU-level bit allocation. For Robot featuring large flat areas and simple edges, insufficient valid edge information degrades adaptive bit allocation performance.

These results reveal the limitations of the current JND-PRC model when handling screen videos with low motion, low texture, and simple content. In contrast, the spatiotemporal feature model proposed in [27] leverages multi-frame temporal correlation analysis and exhibits advantages in processing such screen videos. In future work, we plan to introduce the temporal feature model from [27] as a preprocessing stage and integrate it with our JND model in the perceptual optimization phase. This is expected to achieve further performance improvements in a broader range of SCV scenarios. We will also migrate the optimized model framework to the VVC video-coding standard for performance verification and generalization enhancement.

## 5. Conclusions

This paper targets the application scenarios of screen content videos (SCVs) captured by visual sensors and image sensor networks, and proposes a perceptual rate control model for screen content videos based on the just-noticeable distortion (JND) of the human visual system. The edge model is used to extract the edge of the screen content video collected by sensors, and the edge is reconstructed according to the edge brightness adaptation, contrast masking effect and edge structure sensitivity, and the perceptual features of the video are extracted. The extracted edge factors and the final JND model are used to classify the perception complexity and allocate the target bit at the frame level and CTU level. Then, based on the edge similarity between the reference frame and its reconstructed video frame, the perceptual rate control model is constructed under the condition of the JND constraint. Extensive experimental results demonstrate that the proposed algorithm achieves superior rate-distortion performance for screen content video coding.

In our future work, we will focus on the following four aspects: (1) introduce inter-frame perception weights to specifically handle scenarios such as screen scrolling and window sliding, thereby suppressing encoding flicker artifacts; (2) quantitative computational complexity analysis will be conducted in our future research to verify its feasibility for deployment on low-power sensing terminals; (3) optimize the limitations of the JND model in scenarios with low motion, low texture, and simple content; (4) migrate the proposed algorithm to the new VVC coding framework to verify its universality.

## Figures and Tables

**Table 1:** SCC test conditions.

Categories	Sequences	Resolution	Frame Rate (Hz)	Frames To-Be-Encoded
TGM	WebBrowsing	1280 × 720	10	160
	Programming	1280 × 720	60	160
	flyingGraphics	1920 × 1080	60	160
	Console	1920 × 1080	60	160
	Desktop	1920 × 1080	60	160
M	MissionControlClip3	1920 × 1080	60	160
	MissionControlClip2	2560 × 1440	60	160
	BasketballScreen	2560 × 1440	60	160
A	Robot	2560 × 1440	30	160

**Table 2:** Bit rate mismatch comparison of proposed algorithm with SCM8.0.

Cat.	Sequence	QP	Target (Kbps)	SCM-NH (%)	JND-NH (%)	SCM-H (%)	JND-H (%)	SCM-A (%)	JND-A (%)
TGM	Web browsing	22	61.366	1.139	0.272	0.241	1.101	0.002	0.282
		27	48.479	6.929	2.216	1.958	0.006	1.549	0.464
		32	37.187	7.316	3.698	1.799	0.013	0.024	0.261
		37	28.317	8.251	2.878	3.231	0.238	0.028	0.053
	Programming	22	5004.618	0.046	0.124	0.216	1.171	0.098	0.063
		27	2611.953	0.008	0.187	16.641	0.271	5.593	0.387
		32	1302.384	0.077	0.405	1.432	1.527	1.389	1.236
		37	666.972	0.217	0.443	0.950	0.725	1.121	0.807
	Console	22	5051.949	4.894	2.333	1.186	2.150	1.501	1.280
		27	4007.562	3.085	0.043	0.975	0.123	3.732	1.489
		32	3099.228	3.933	0.450	1.554	0.219	0.110	0.255
		37	2356.314	2.405	0.173	0.203	0.100	0.503	0.522
	Desktop	22	1128.852	1.974	4.873	4.474	16.268	0.103	6.757
		27	1064.652	1.417	3.748	1.690	14.484	0.522	5.511
		32	966.177	0.671	3.783	0.376	13.134	0.669	4.387
		37	923.121	0.093	3.590	0.203	10.798	0.673	3.443
M	MissionControlClip3	22	1867.605	0.004	3.196	1.027	0.707	3.927	0.073
		27	1086.744	0.007	1.771	0.084	0.708	0.388	0.475
		32	678.627	0.005	1.568	0.204	0.712	0.375	0.528
		37	446.895	0.005	1.614	0.281	0.922	0.559	0.815
	MissionControlClip2	22	8885.331	4.238	2.176	8.871	2.872	11.800	3.163
		27	5056.632	5.806	1.661	0.062	2.221	0.038	0.231
		32	2759.331	2.406	0.003	0.025	1.625	0.071	0.084
		37	1502.841	0.001	0.035	0.123	0.712	0.131	0.489
	BasketballScreen	22	5629.722	0.075	3.270	2.694	0.290	3.849	1.001
		27	2734.356	0.015	1.666	0.072	0.652	1.413	0.347
		32	1464.459	0.120	1.428	0.143	0.838	0.592	0.750
		37	853.149	0.129	0.888	0.081	0.734	0.008	0.884
A	Robot	22	5111.223	0.006	0.083	0.029	0.090	0.030	0.062
		27	1697.786	0.006	0.154	0.065	0.021	0.065	0.072
		32	596.295	0.010	0.030	0.169	0.200	0.167	0.362
		37	242.339	0.015	0.127	0.411	0.199	0.406	0.570
Average				1.728	1.528	1.608	2.370	1.295	1.159

**Table 3:** R-D performance comparison of the proposed algorithm with SCM 8.0 under LDB.

Cat.	Sequence	JND-PRC-NH vs. SCM-8.0-NH		JND-PRC-H vs. SCM-8.0-H		JND-PRC-A vs. SCM-8.0-A		Average	
		BD- BR (%)	BD- YPSNR (dB)	BD- BR (%)	BD- YPSNR (dB)	BD- BR (%)	BD- YPSNR (dB)	BD- BR (%)	BD- YPSNR (dB)
TGM	Web browsing	−46.96	7.25	−30.13	3.97	−31.44	4.06	−30.53	4.42
	Programming	−30.66	2.01	−26.62	1.75	−25.99	1.60		
	Console	−6.69	1.24	−3.14	0.69	−6.22	1.36		
	Desktop	−77.88	11.09	−27.65	8.75	−46.63	9.26		
M	MissionControlClip3	−47.94	5.90	−45.18	4.84	−42.21	4.42	−32.39	3.08
	MissionControlClip2	−6.83	0.45	−13.00	0.90	−13.09	0.92		
	BasketballScreen	−46.21	4.19	−40.01	3.20	−36.93	2.86		
A	Robot	−15.60	0.44	−9.97	0.28	−9.59	0.27	−11.65	0.33
Average		−34.85	4.07	−24.46	3.05	−26.51	3.09	–	–

**Table 4:** R-D performance compared with other literature under LDB.

SCV Sequence	Yang et al. [30] vs. SCM-8.0-H		Lin et al. [27] vs. SCM-8.0-H		JND-PRC vs. SCM-8.0-H	
	BD-YPSNR (dB)	BD-BR (%)	BD-YPSNR (dB)	BD-BR (%)	BD-YPSNR (dB)	BD-BR (%)
BasketballScreen	0.84	−9.97	2.02	−30.35	3.20	−39.94
Console	0.79	−4.25	1.65	−9.93	0.68	−3.09
Desktop	0.65	−4.16	3.10	−27.57	8.71	−27.47
MissionControl2	0.33	−4.87	1.41	−20.54	0.89	−12.95
MissionControl3	1.45	−14.17	2.61	−35.24	4.82	−45.02
Programming	1.20	−19.07	1.96	−25.21	1.74	−26.53
Robot	0.66	−21.37	0.49	−17.68	0.27	−9.23
Web browsing	0.86	−6.95	1.77	−18.61	3.89	−29.47
Average	0.85	−10.60	1.88	−23.14	3.03	−24.21

## Data Availability

The datasets analyzed in this study were obtained by “A frame-level rate control algorithm for screen content coding” [31].
